# The sex life of male patients with cirrhosis and its organic factors: What we have got so far?

**DOI:** 10.1371/journal.pone.0280915

**Published:** 2023-02-02

**Authors:** Darmadi Darmadi, Cennikon Pakpahan, Riska Habriel Ruslie, Bella Amanda, Raditya Ibrahim

**Affiliations:** 1 Faculty of Medicine, Department of Internal Medicine, Universitas Sumatera Utara, Medan, Indonesia; 2 Faculty of Medicine, Andrology Study Program, Universitas Airlangga, Surabaya, Indonesia; 3 Faculty of Medicine, Department of Biomedical Sciences, Universitas Airlangga, Surabaya, Indonesia; 4 Faculty of Medicine, Department of Child Health, Universitas Prima Indonesia, Medan, Indonesia; University Campus Bio-Medico di Roma, ITALY

## Abstract

**Objective:**

The purpose of this study was to examine the sex lives of male cirrhotic patients organic factors affect them.

**Method:**

We did an observational study of 220 cirrhotic men’s satisfaction with their sexual lives. Assessment of sex satisfaction was carried out using the New Sexual Satisfaction Scale. The frequency of intercourse and masturbation was estimated. Then, the levels of albumin, total bilirubin, vitamin D, and sex steroids were examined. Ascites and sarcopenia subgroups of our patients were stratified.

**Results:**

Along with sex steroids, albumin, total bilirubin, and vitamin D all had an impact on how satisfying sex was (r = 0.22), (r = 0.24), and (r = −0.17) affected sex satisfaction. There were strong positive correlations between vitamin D (r = 0.33), albumin (r = 0.59), and free testosterone, along with a negative correlation between total bilirubin (r = −0.63) and free testosterone. An imbalance in sex steroid levels was observed, leading to decreased frequency of intercourse(p < 0.0001), weakened erections(p < 0.0001), and reduced quality of orgasm (p < 0.0001). Significant new sex behavior changes were found, such as an increase in masturbation. Physical limitations such as ascites and sarcopenia also impacted the decreasing sex life.

**Conclusion:**

The sex life of cirrhotic men is affected. The decrease in the frequency of intercourse and sexual satisfaction is noticeable in male patients and leads to increased masturbation. Free Testosterone, vitamin D, albumin, and bilirubin play role in their sex life. In addition, ascites and sarcopenia not only affect their sex life but also hamper the quality of their well-being.

## Introduction

Liver cirrhosis is a major health issue worldwide. From 1990 to 2017, the prevalence of liver cirrhosis grew globally by 74.53%. The Caribbean and Latin America have seen the biggest increases. Alcohol consumption, Non-alcoholic steatohepatitis (NASH), hepatitis B (HBV), and hepatitis C (HCV) among others, are common causes of liver cirrhosis [1]. A total of 54.3% of deaths from cirrhosis are caused by hepatic cirrhosis, which is the main cause of liver-related deaths in Asia [2]. The global mortality rate has increased from 676,000 in 1980 to over 1 million in 2010 [3]. Infection, gastrointestinal bleeding, hepatic encephalopathy, hepatorenal syndrome, and ascites are only a few of the serious morbidities associated with liver cirrhosis. The infection affects about 20.6% of people with liver cirrhosis [4]. In addition to hurting patient’s sexual function and quality of life, liver cirrhosis also places a heavy burden on society and the families of those [5] who are affected.

Sexual function is also affected in patients with liver cirrhosis. In males, penile erection is the most prominent deteminant of sexual function. Males with liver cirrhosis are at risk of erectile dysfunction. Erectile dysfunction is an inability to attain or maintain a penile erection of sufficient quality to permit satisfactory sexual intercourse. Erectile dysfunction itself affects health-related quality of life in males. According to a study by Paternostro et al., erectile dysfunction was observed in 63.8% of males with liver cirrhosis. Most of them had erectile dysfunction that was mild to severe. The severity of liver dysfunction was related to erectile dysfunction [6]. 61% of participants in a different study on people with alcoholic cirrhosis reported having a sexual dysfunction. Erectile dysfunction and reduced sexual desire were the most common symptoms reported. Subjects without cirrhosis experienced a considerable reduction in symptoms. Hypogonadism and symptoms of feminization, testicular atrophy, low testosterone levels, decreased libido, infertility, diminished secondary sex hair, and gynecomastia were other important changes seen in cirrhosis patients [7–9].

A possible cause of decreased sexual function in patients suffering from liver disease is an abnormality of the hypothalamic-pituitary-gonadal axis [10,11]. In sexuality and reproduction, Sex enhancers play a crucial role. A previous study reported improved erectile function in subjects with end-stage liver diseases after liver transplantation. After the surgery, the status of hypogonadism was also addressed. This supports that liver function is associated with sexual function [12,13].

Study regarding sexual life in males with liver cirrhosis in Indonesia is scarce. In this study, we sought to ascertain the sexual function of male liver cirrhosis patients. It is important since sexual life plays a role in improving the patient’s quality of life.

## Materials and methods

The participants in this retrospective study are males with liver cirrhosis. The study was conducted at Haji Adam Malik General Hospital Medan, Indonesia, from May 2021 to June 2022. Consecutive sampling was used to collect subjects. Male patients with liver cirrhosis who were willing to participate in the trial and were at least 18 years old met the inclusion criteria. The diagnosis of liver cirrhosis was established using ultrasonography and FibroScan®. Additionally, we divided the patients into groups based on comorbidities, clinical signs such as ascites and sarcopenia, and a Child-Pugh Class evaluation of the severity of cirrhosis. Concurrent sexual dysfunction that existed before the diagnosis of liver cirrhosis was the exclusion criterion. The Institutional Review Board of Universitas Sumatera Utara approved this study with approval letter number 153/KEP/USU/2021.

Each subject provided demographic and medical information. We used New Sexual Satisfaction Scale (NSSS) by Stulhofer, Busko, and Brouillard [14] to determine the subjects’ sex life. Tahalele confirmed the questionnaire after having it translated into Bahasa Indonesia [15]. Each subject completed the questionnaire twice: first to explain the circumstances before diagnosis and again to explain the condition following the diagnosis of liver cirrhosis. The decline of libido was positive if the patient were not interested in sex (had no sex drive or low sex drive) and was bothered by it. Decrease in orgasms characterized as delayed, infrequent, or absent orgasms–that are much less intense, following adequate sexual stimulation and arousal. The patient’s erection quality was measured using the erection hardness score (EHS). Eagle Biosciences’ chemiluminescent microparticle immunoassay was used for liver function tests (bilirubin, albumin), and its ELISA for vitamin D was used for testing (Nashua, NH, USA). We determined levels of estradiol, free testosterone, total testosterone, and sex hormone-binding globulin from each patient. Peripheral blood was utilized as the sample in an enzyme-linked immunosorbent test (ELISA) kit from (Roche Diagnostics Ltd., Shanghai, China).

We presented qualitative data in frequency and percentage. A normality test was performed using quantitative data. If the data were normally distributed, it would be presented as mean and standard deviation. If not, it would be shown as the median and the range of values. A Chi-square test was utilized to determine the association between sex life and pre and after-diagnosis with cirrhosis.

If the data were normally distributed, an independent t-test was used to assess the differences in sexual satisfaction and sex steroid levels between groups. If the data were not normally distributed, the Mann-Whitney test was used instead. Meanwhile, in the test of differences between more than two groups, one-way ANOVA was used if the data were normally distributed, and Kruskal Wallis if the data were not normally distributed. In addition, if the data were normally distributed, we used the Pearson correlation test to examine the correlation between variables; otherwise, we used the Spearmen test. Graph Pad Prism 9 software was used for statistical analysis, and a p-value of <0.05 was considered significant.

## Results

### Demography and laboratory characteristics

A total of 220 men with a mean age of 56.07 (±63.65) years were involved in this study. According to this research, cirrhosis is more common in late adulthood. Cirrhosis was also often accompanied by clinical symptoms such as ascites (91%) and sarcopenia (81.4%). According to the Child-Pugh Class, the severity of the patients who participated in this study was divided. The majority of subjects in this study were in the severe groups (CPC-class C) based on the classification. As many as 161 (73.18%) patients belonged to class C. Hepatitis B and C, chronic kidney disease, congestive heart failure, TB, ischemic stroke, and diabetes mellitus were among the additional comorbidities present in the study’s participants. Hepatitis B, one of the risk factors for cirrhosis, was reported as the highest number of comorbid conditions (38.6%), followed by hepatitis C (10.6%).

We performed laboratory tests to investigate the patient’s complaints. They included vitamin D, total cholesterol, bilirubin, and albumin among other markers. In addition, sex hormones that regulate liver metabolism were investigated. Sex hormone-binding globulin (SHBG), free testosterone, total testosterone, and estradiol are some of these (Table 1).

**Table 1:** Demographic and laboratory characteristics.

Variables	Result
Age	56.07 (±63.65)
Ascites Yes No	20 (9%) 200 (91%)
Sarcopenia Yes No	41 (18.6%) 179 (81.4%)
Child-Pugh Class (CPC) Class A Class B Class C	23 (10.45%) 36 (16.36%) 161 (73.18%)
Comorbid Diabetes Mellitus Chronic Kidney Disease (CKD) Hepatitis B Hepatitis C Congestive Heart Failure (CHF) Tuberculosis and Lung Disease Ischemic Stroke	8 (3.6%) 4 (1.8%) 85 (38.6%) 23 (10.6%) 6 (2.7%) 3 (1.4%) 4 (1.8%)
Sex Satisfaction Score	33 (30, 39)
Sex Score Satisfy (Score > 60) Non-satisfy (Score < 60)	20 (9%) 200 (91%)
Erection Hardness Score 1 2 3 4	44 (20%) 135 (61.36%) 21 (9.64%) 20 (9%)
Vitamin D (ng/ml)	20 (18, 50)
Total cholesterol (mg/dl)	140 (130, 155)
Estradiol (pg/ml)	158 (145, 174)
Free Testosterone (ng/dl)	0.825 (0.75, 1.388)
Total Testosterone (nmol/l)	6.8 (6.4, 10.4)
SHBG (nmol/l)	98 (88, 108)
Total Bilirubin (mg/dl)	3.990 (3.400, 4.400)
Albumin (g/dl)	2.450 (2.300, 2.695)

### Sex life parameters before and after cirrhosis diagnosis

Utilizing the NSSS, the satisfaction level was measured (New Sexual Satisfaction Scale). The average score in this study was 33 (30, 39). This showed low levels of satisfaction in this study. Only 20 (9%) of the patients reported being satisfied with their sexual life if the cut-off is raised to >60. In addition to testing other aspects of sexual function, such as firmness of the erection, 135 (61.36%) participants said their penis was too weak to penetrate. This contributed to a low level of sexual satisfaction (Table 1).

**Fig 1:**
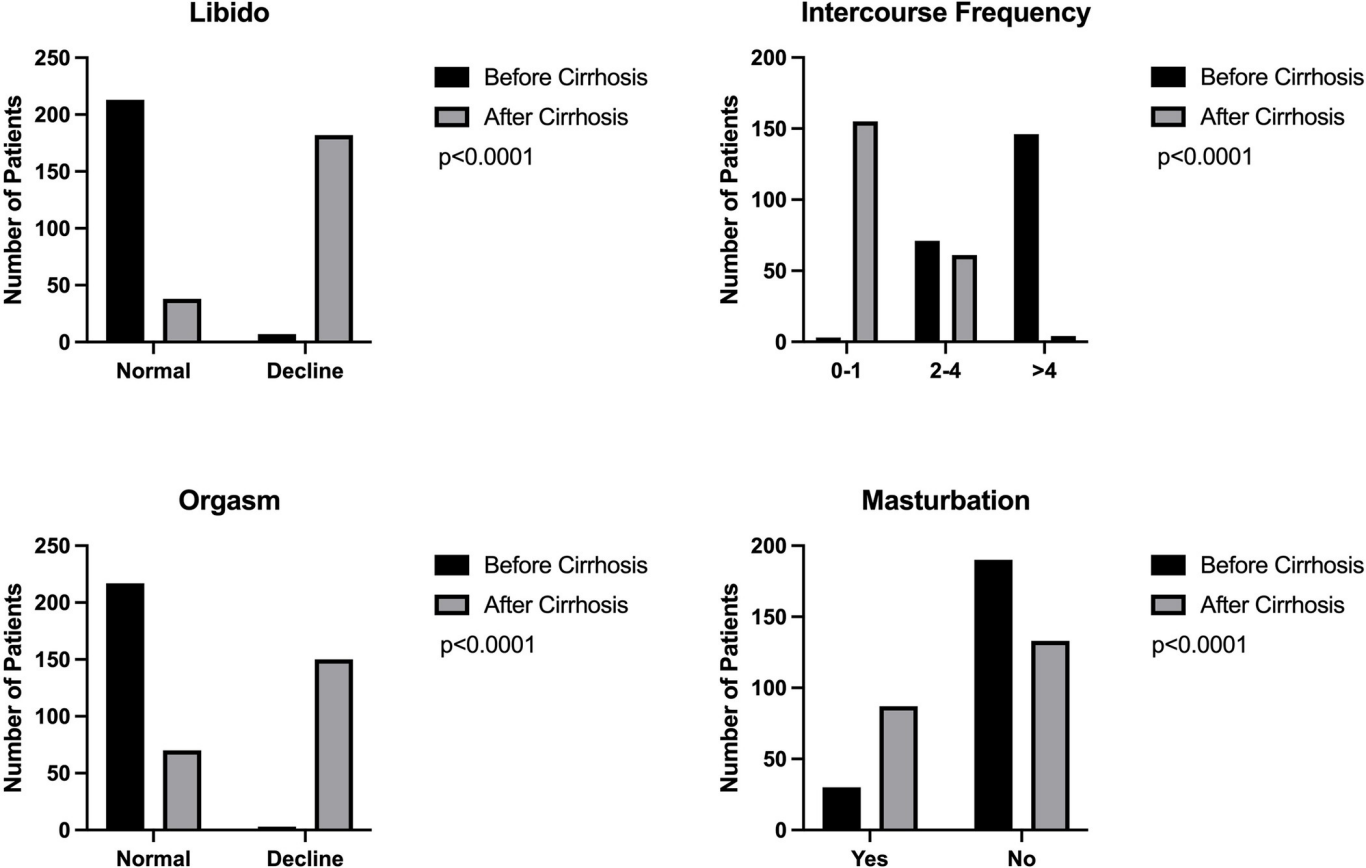
Sex life parameters before and after cirrhosis diagnosis.

The findings of the sex life factors we assessed revealed sizable variations before and after the cirrhosis diagnosis (Fig 1). There was a very significant difference in libido before and after being diagnosed with cirrhosis. Before being given a cirrhosis diagnosis 213 (96.8%) patients had normal libido; after receiving that diagnosis, that number dropped to 38 (17.3%) (p < 0.0001). Other than libido, sexual activity reduced significantly (p < 0.0001) in frequency. A total of 146 (66.36%) reported regular intercourse more than four times in a month before the diagnosis of cirrhosis compared to 4 (1.83%) after the diagnosis. Of course, this also caused patients to experience fewer orgasms in their sex life. There was a significant difference; initially, 217 (98.6%) patients experienced an orgasm and it decreased to only 70 (31.8%) (p < 0.0001). The patient’s incapacity to engage in intercourse was replaced with other types of sexual behavior, such as masturbation, which was supported by a lower frequency and EHS. Before the patient was diagnosed with cirrhosis, only 30 (23.7%) of them masturbated. However, 87 patients (39.5%) significantly increased their masturbating after receiving a diagnosis (p < 0.0001).

### Sex steroid and sex score evaluation in sub-group analysis

**Fig 2:**
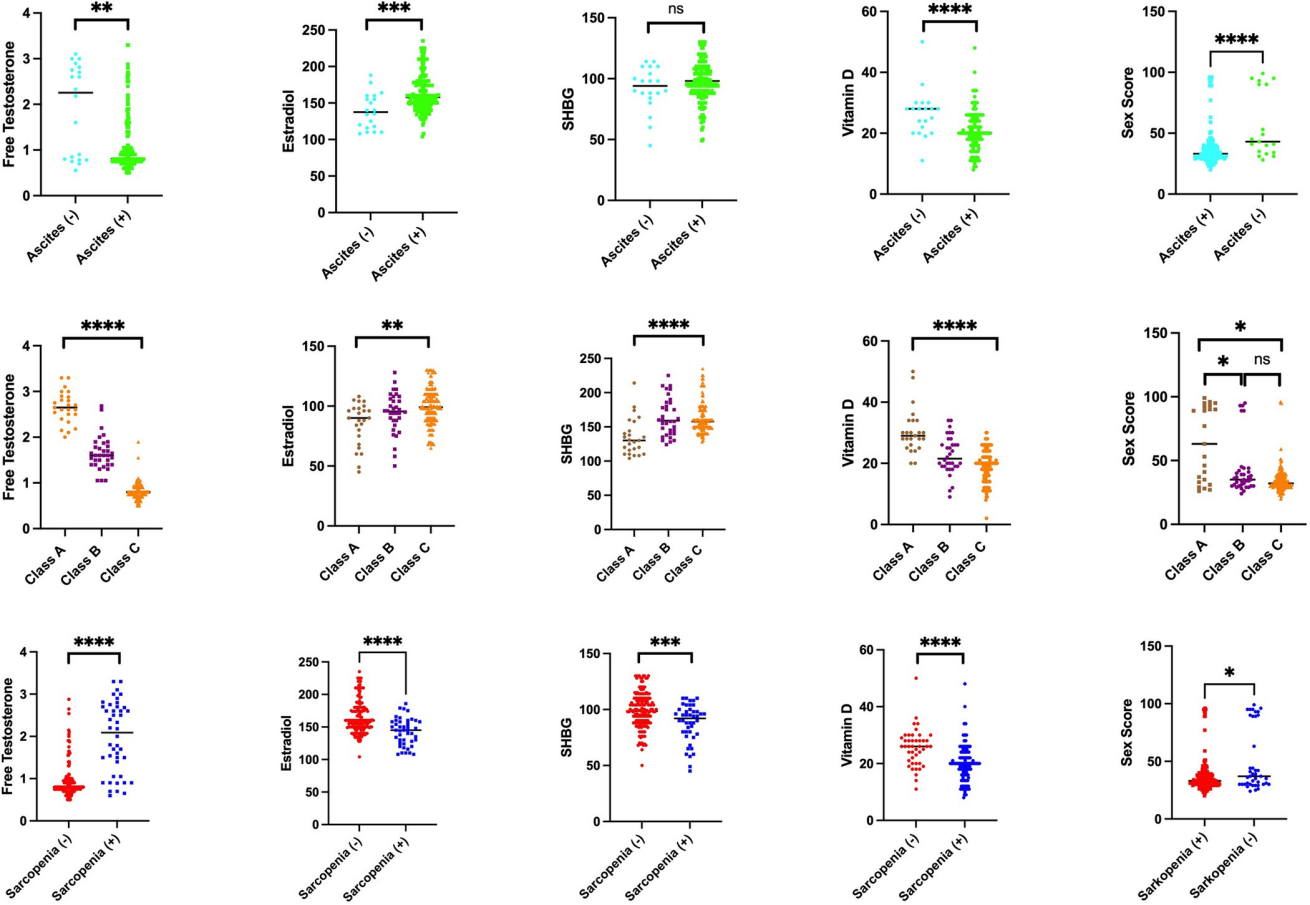
Sex steroid and sex score evaluation in sub-group analysis.

We stratified and then analyzed three clinical features: ascites, Child-Pugh Class, and sarcopenia. These three were compared to laboratory variables including sex hormones and sex enjoyment (Fig 2). Ascites were associated with low levels of free testosterone (p = 0.0015) and estradiol (p = 0.0003), but not with SHBG (p = 0.3036). Patients with ascites tended to be less satisfied than those without ascites at the time of the test on sexual satisfaction,(p < 0.0001). In the Child-Pugh Class group, there were significant differences between classes in free testosterone (p < 0.0001), estradiol (p < 0.0001), and SHBG (p < 0.0016). In comparison to classes A and B, Class C had lower free testosterone, greater estradiol levels, and higher SHBG. There was a significant difference between the three when analyzing sexual pleasure (p = 0.0002); however, when the post-hoc test was run, the difference was only shown between classes A vs. B (p = 0.0186) and A vs. C (p < 0.0001) but not B vs. C (p = 0.071). Free testosterone, estradiol, and SHBG levels were shown to be substantially correlated with the parameter of sarcopenia (p < 0.0001), estradiol (p < 0.0001), and (p = 0.0002, respectively). Then the sex satisfaction between the two was significantly different (p = 0.036). In individuals with ascites, sarcopenia, and Child-Pugh Class C sex steroid circumstances such as decreased free testosterone and elevated estradiol and SHBG may have an impact on the consistent outcomes between these three variables for lower sexual enjoyment.

### Correlation between laboratory parameters and sex score

The relationship between sex satisfaction scores and factors such as age, vitamin D, albumin, total cholesterol, total bilirubin, estradiol, SHBG, free testosterone, and total testosterone was examined by correlation analysis. From several laboratory parameters examined, vitamin D, total bilirubin, albumin, free testosterone, and total testosterone were significantly correlated (Table 2).

**Table 2:** Matrix correlation between the independent variable (laboratories) vs. sex score.

Dependent Variable	Independent Variable								
	Age	Vitamin D	Albumin	Total Cholesterol	Total Bilirubin	Estradiol	SHBG	Free Testosterone	Total Testosterone
**Sex Score (r)**	0.09	0.22	0.24	0.04	−0.17	−0.10	−0.05	0.22	0.20
**p-value**	0.181	0.0009***	0.0003***	0.446	0.0096**	0.134	0.390	0.0008***	0.0027**

*p-value significance with the spearman correlation test.

**Fig 3:**
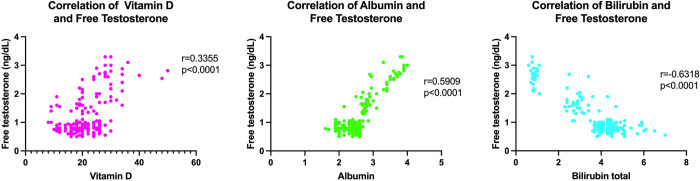
Correlation between the independent variable (vitamin D, albumin, and total bilirubin) vs. free testosterone.

We examined the relationship between vitamin D, albumin, total bilirubin levels, and free testosterone after discovering that these three variables were substantially connected with sexual pleasure ratings (Fig 3). The test results showed that vitamin D and albumin had a positive correlation (p < 0.0001), while total bilirubin had a negative correlation with free testosterone (p < 0.0001). We assume that there is a significant relationship between the sex satisfaction scores and the variables (vitamin D, albumin, and bilirubin) via the free testosterone pathway.

## Discussion

Cirrhosis is a chronic liver disease that is irreversible and progressive. This illness results in a wide range of complications and possesses high medical cost [16]. Cirrhosis is caused by several etiologies including viral hepatitis. The mortality rate of this disease is significant and dominated by females [17,18]. The median survival of patients with cirrhosis is seeaid to be 12 years, and the clinician is responsible for maintaining the patient’s quality of life as long as he survives, including his sex life. This is also important along with the prevention of mortality and morbidity [19].

Patients with cirrhosis have been noted to have low sexual quality. In our study on 220 male cirrhotic patients, 91% of patients reported being unsatisfied with their sexual life, scoring an average of 33 on the “New Sexual Satisfaction Scale (NSSS)” (30, 39). In addition, 20% complained of low erection strength equal to the first degree of EHS and 61.36% with the second degree of EHS. This shows that almost all of the patients were unable to approach their partners. The same finding was also reported by Kim et al. [20] and Paternostro et al. [6]. According to this research, if erectile dysfunction is present together with concomitant conditions like hypertension and diabetes mellitus, its prevalence will rise in cirrhotic patients. While in this study, some were reported to have comorbidities such as diabetes mellitus, CKD, and CHF, which further worsened the patients’ erectile quality.

In addition, this retrospective study found that after receiving a cirrhosis diagnosis, their libido, orgasm quality, and frequency of sex reduced dramatically (p < 0.0001). Interestingly, masturbation activity increased after being diagnosed with cirrhosis (p < 0.0001). However, there is still no research reporting the decline in orgasm and the frequency of intercourse [21]. This decline in libido is comparable to the study of Neon, Billington, and Cong. We predicted that patients who experience a decline in libido and orgasm would engage in fewer sexual encounters; in addition, patients who perceive a decline in the quality of sexual relationships tended to be pessimistic and depressed. This is in parallel with the study of Fidan et al. [22] who reported that depression and sexual quality are influencing each other. It is interesting to note that depression in cirrhotic patients was closely correlated with CPC-measured disease severity. Logically, a patient would decide to have fewer sex acts to minimize dissatisfaction with both himself and his partner, and as a result, would seek out alternate sexual activities like masturbation.

We suspected the clinical presentation of cirrhotic patients to be a risk factor for sexual dysfunction. Sexual satisfaction levels dropped considerably between the positive and severe CPC subgroups for the three clinical symptoms, ascites, sarcopenia, and severity. This might be due to a decrease in sex steroids that have a role in sexual function, accompanied by an increase in estrogen and SHBG [23]. The group with positive ascites, sarcopenia, and CPC-class C tended to have low free testosterone and high estrogen levels as well as SHBG in this sub-group analysis. In addition, somatization factors and physical limitations, such as ascites and sarcopenia, maybe the reason for the decrease in sex satisfaction scores. Additionally, a decline in self-esteem brought on by actual physical changes can result in sadness, tension, and partner disputes [21].

The pituitary-gonadotropic axis in patients with cirrhosis is indeed impaired. The disrupted sex hormone profile in cirrhosis may be brought on by metabolic issues with portosystemic hemodynamics (hypoestrogenism and elevated SHBG), or by the effects of drugs like spironolactone [21]. Cirrhosis has been reported to play an important role in pituitary luteinizing hormone (LH) suppression, which results in decreased circulating testosterone [24,25]. It is consistent with the findings of this study that both total and free testosterone tended to decline. In addition, Burra et al have shown in their study that prolactin in cirrhotic patients is increased, suppressing the production of gonadotropin-releasing hormone (GnRH) in the pituitary gland [24]. Another process that increases peripheral testosterone conversion to estrogen is blood flow shunting from peripheral tissue to the liver [26]. Men with cirrhosis produce an amount of testosterone by peripheral conversion from androstenedione, which accounts for about 15% of total testosterone [27].

The analysis of the relationships between laboratory data, including sex steroids (free testosterone and total testosterone), vitamin D, albumin, and total bilirubin, which all significantly correlated with the score of sexual pleasure, is what makes this study intriguing. Hypoalbumin was reported as an independent factor (r = 0.24, p = 0.0003) in this study, which is in line with the result from studies conducted by Kim et al. [20] and Toda et al. [28]. The ratio of albumin-bound to free testosterone, which is linked to sexual desire and sleep-related erections, can be impacted by decreased albumin synthesis, which can also limit the patient’s capacity to move around physically [29]. Additionally, our study found that free testosterone and vitamin D both had favorable correlations with sex scores that have a direct impact on sexual function (r = 0.335, p < 0.0001) and sex scores (r = 0.22, p = 0.0009). The association between vitamin D deficiency and hypogonadism has been widely reported. The claim about the Chinese population is supported by research by Wang et al. The mechanisms that play a role in this condition are adiposity and insulin resistance [30]. The involvement of vitamin D in Leydig cells is another way. Testosterone production in Leydig cells is under LH control, which depends on the intracellular calcium ion concentration. The calcium level-dependent LH response may be modified by vitamin D [31].

Meanwhile, Park et al. [32] reported a negative correlation (r = −0.17, p = 0.0096)between total bilirubin and testosterone. Elevated bilirubin is thought to be the mechanism responsible for chronic low-grade inflammation and oxidative damage. A subclinical inflammatory condition, which frequently manifests in testosterone deprivation, is brought on by inflammation by stimulating the release of pro-inflammatory cytokines tumor necrosis factor, and interleukin [33]. Another mechanism is insulin resistance and metabolic abnormalities may lead to testosterone deficiency [34,35].

Improving the quality of life of patients with cirrhosis is still complicated; several studies reported that this irreversible disorder is better treated with liver transplantation. The patient’s quality of life including sexual life will be improved by the transplant. However, because sex life is influenced by both organic variables, such as sex hormones, and psychogenic factors, such as depression, sex life sometimes does not immediately benefit from post-transplantation. Depression in post-transplant patients is sometimes not resolved, which is negatively correlated with their sex life [24]. On the other hand, transplant treatments are not always simple to undertake due to the accessibility of services that perform these procedures and the availability of donors, particularly in middle-low-income nations. For cirrhotic patients to continue having sex with a partner, sensate focus (sex without penetration) is still a good idea [36].

This study had several limitations; including missing data on a history of alcohol, consumption, the use of medicines like propranolol and spironolactone, prolactin levels, long-term cirrhosis symptoms, and the probability of depression in the patient. In addition, information about sex life before the diagnosis of cirrhosis relies on recall, which is undoubtedly prone to bias. We also believe that this study’s data is quantitative, whereas qualitative information about sex life may be more insightful and detailed. Lastly, most subjects in this study were grouped in Child-Pugh C class which means they had severe liver dysfunction. This issue may interfere our study results due to impact of liver dysfunction severity and erectile dysfunction. Therefore, it is necessary to encourage similar research by relying on qualitative data to get a different perspective on sex life in cirrhotic patients.

## Conclusions

One of the most important factors to take into account when evaluating a person’s quality of life, especially those with cirrhosis, is their sexual life. Alterations in the Hypothalamus-Pituitary-Gonadal axis cause disturbances in levels of sex steroids such as testosterone. The hormone testosterone is the cause of human sex. This study suggests that an imbalance in sex steroid levels contributes to lower sexual activity, weakened erections, and poorer quality orgasms. As a result, significant new sex behavior changes were found, such as an increase in masturbation. Physical constraints such as ascites and sarcopenia, in addition to sex steroids, have an impact on diminishing sex life. What is new in this study is that vitamin D, albumin, and total bilirubin affect the sex life of cirrhotic patients. The patient’s quality of life can be enhanced by liver transplantation, a therapeutic option that can resolve sex life. While waiting for a transplant, a sensate focus needs to be directed to maintain the sex life of patients with cirrhosis.
